# Supplementary figures and images for: Effects of vermicompost and lime on acidic soil properties and malt barley (Hordeum Distichum L.) productivity in Mecha district, northwest Ethiopia
Source: PLoS One. 2024 Dec 6;19(12):e0311914. doi: 10.1371/journal.pone.0311914 (PMC11623467; doi:10.1371/journal.pone.0311914)

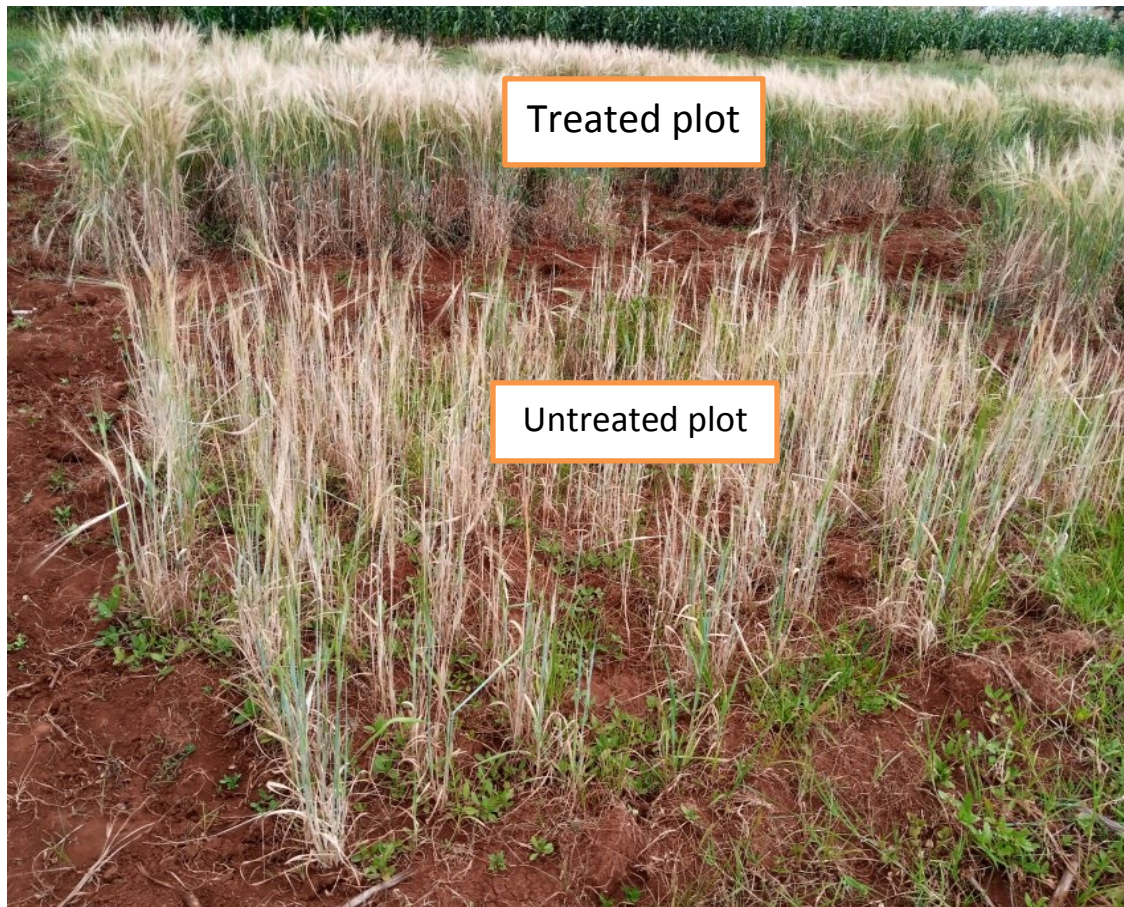

**S1 Fig.**

Supplement: S1 Fig — (PDF) [file pone.0311914.s001.pdf]

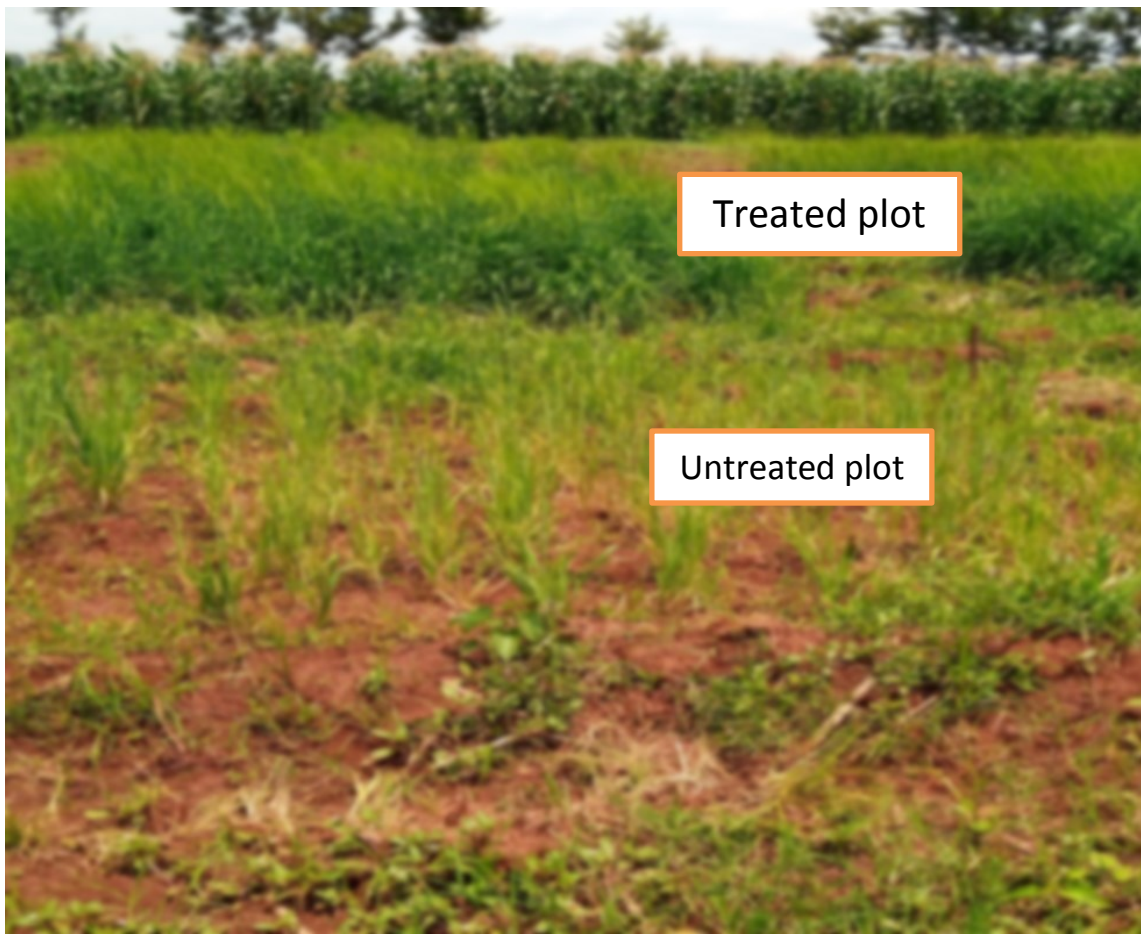

**S2 Fig.**

Supplement: S2 Fig — (PDF) [file pone.0311914.s002.pdf]
