# Supplementary material for: Effects of vermicompost and lime on acidic soil properties and malt barley (Hordeum Distichum L.) productivity in Mecha district, northwest Ethiopia
Source: PLoS One. 2024 Dec 6;19(12):e0311914. doi: 10.1371/journal.pone.0311914 (PMC11623467; doi:10.1371/journal.pone.0311914)
Supplement: Attachment — Submitted filename: COMMENTS...pdf [file pone.0311914.s003.pdf]

Comments

I have gone through the exhaustive article on "**Effects of vermicompost and lime on soil properties and malt barley (Hordeum distichum L.) productivity on acidic soils of Mecha district, northwest Ethiopia**" In the article, the previous literature is well-documented by the authors. However, the following research articles are needed to be cited in the introduction and methodology section.

.

1. DOI: 10.1007/s11104-023-06016-4
2. DOI: 10.3390/microorganisms10102078
3. DOI: 10.4236/ojss.2018.82007
