# Supplementary material for: Effects of vermicompost and lime on acidic soil properties and malt barley (Hordeum Distichum L.) productivity in Mecha district, northwest Ethiopia
Source: PLoS One. 2024 Dec 6;19(12):e0311914. doi: 10.1371/journal.pone.0311914 (PMC11623467; doi:10.1371/journal.pone.0311914)
Supplement: Attachment — Submitted filename: Reviewers response.docx [file pone.0311914.s004.docx]

**Title:** Please replace the word “of’ with the word ‘in’ before Mecha

**Abstract:**

Line 16: replace the word “of’ with the word ‘in’ before Mecha

Line 18: make the word arrange in past form

Please write clearly, about tons and ton; Use the elaborate form of VC and L

**Keywords:** Use more appropriate words

**Introduction:**

- In the first sentence, use a conjunction to complete the sentence
- In the second sentence add an article before the word Ethiopian and leaching
- In the third sentence, ‘acidic (pH 4.1-5.5) soils’ write in nature instead of soils.
- The first sentence and last sentence of first para (line 33-38) is almost same. Make it in one sentence.
- Line 39: add scientific name of crop.
- “Although barley is considered to be the fifth-most common, a widely farmed crop in Ethiopia's highlands, it is extremely susceptible to soil acidity” the sentence is not clear, make it clear and simple.
- Please remove the decimal and give a round figure (Line 42).
- Always maintain the same unit
- Line 47: Please use recent information
- Line 48-49: Rewrite the sentence considering the article, tense, and punctuation
- Line 53 please use recent data
- Line 54-56. Check these sentence “Besides, vermicompost is enriched with soil microbiota such as nitrogen-fixing and phosphorus-solubilizing organisms and provides several essential macro and micronutrients become more available in VC [15]. It including the ability to increase soil microbial activity by enhancing oxygen availability, regulating soil temperature, improving porosity, infiltration, and nutrient content, growth promoter and boosting crop yield and quality” and write clearly following grammatical rules
- Line 61: Add an article before the word economically
- Line 64: Mention maximizing instead of maximize
- Line 66: Write have instead of has

**Materials and Methods:**

- Please check the sentence “The study site is located between 11°^”^ 29' 30" N and 11° 33' 0" N latitudes and 37° 07' 0" E and 37° 10' 30" E” longitudes”
- The study was carried out in 2021 and 2022, but the meterological data was given from 2011 to 2020. Please give the data only during the cropping season.

- Convert the word productions to production
- Please rewrite the sentence “The major crops type growing the vicinity are wheat, barley, maize, teff and various types of horticultures”
- Line 101 and 103: Check the unit of bulk density
- Line 114: Use sowing instead of planting as seed was used for crop production
- Please mention the source of N and P fertilizer
- Please explain whether boron fertilizer was used or not because it is related to grain formation
- Line 127: convert the word ground to grinded
- Line 133: Please read the sentence carefully
- Follow the same pattern for writing unit
- Manual separation of the grains from the straws resulted in yield measurements for each plot in tons (kg ha^-1^). Write the sentence clearly
- Check the word ‘hectare’ throughout the manuscript
- Line 148: Unify the unit

**Tables:**

- For better understanding, use the short name of the treatment instead of the percent and rate of vermicompost and lime. Then elaborate on the short name of the treatment in the table footnote
- Synchronize the treatments serials in table-1 with table-3,4 and 6
- Mention the area for vermicompost and lime rate
- Please solve the problem of t/ha, t ha^-1^, ton/ha, t ha^-1^, tons, and ton
- Please check the sentence “Table 2. Characteristics the study soil and vermicompost before planting” and correct the sentence
- Table-1: Explain, for treatment combination, why don’t use 50% sole lime whether use 50% sole vermicompost?; use 150% sole lime but why not 150% vermicompost? also use combination of 150% VC+100 L but why not 150% L+100% VC
- Table-2: BD is appearing very low, whether soil is clay and organic carbon content is very low
- Table-3: Check the *p* vale of available Phosphorus
- Table-6: Please use a round figure for NB and MRR, mentioned GNR in the table caption whether used GNB in the table footnote, write correct one
- Unify the tables heading with the description heading in the result and discussion section
- Please use the same font for footnote

**Result and discussion**

- Please add the field capacity data as irrigation was supplied based on the field capacity
- 183-184 line: C:N and C/N , write this in a uniform pattern
- Please remove clumsiness and duplication during the writing of results and discussion of results. Discuss the results specifically. Omit unnecessary results.
- Please reduce the number of references and use recent references, make space for two or more references in the third bracket
- Make similarity for writing p, ap, phosphorus, and available phosphorus
- Line 264: Place a verb after the reference number 43
- Line 276: Put a comma before respectively
- Line 303: Remove the bracket before 29.63
- Please add the value of the control treatment during the comparison

**Partial budget analysis:**

- First sentence (line 326-329), please read the sentence carefully and rewrite the sentence for better understanding
- Line 330: Please use a round figure for data value
- Please check the net benefit value described in the abstract, partial budget analysis, and conclusion chapters and make uniform

**Conclusion:**

- Consider the same pattern for RL, LR, and L
- Line 344: lime compost? not clear

**References:**

References were written whimsically. Rewrite the reference chapter and follow a pattern for all reference.

Overall comments:

English of the manuscript is poor. Improvement is required. Please go through many rounds of editing, taking into account typos and grammatical problems, before submitting the updated work.

**Following the addition of the aforementioned remarks, the manuscript can be approved.**
