# Supplementary material for: Effects of vermicompost and lime on acidic soil properties and malt barley (Hordeum Distichum L.) productivity in Mecha district, northwest Ethiopia
Source: PLoS One. 2024 Dec 6;19(12):e0311914. doi: 10.1371/journal.pone.0311914 (PMC11623467; doi:10.1371/journal.pone.0311914)
Supplement: Attachment — Submitted filename: Response to the Reviwers.docx [file pone.0311914.s005.docx]

Dr. Ravinder Kumar,
Academic Editor, PLOS ONE

**Subject: Response to Reviewers Comments**

Dear Dr. Ravinder Kumar,

We want to thank you for allowing us to resubmit the revised manuscript entitled " **Effects of vermicompost and lime on soil properties and malt barley (*Hordeum distichum* L.) productivity on acidic soils in Mecha district, northwest Ethiopia** [PONE-D-24-13572] for publication in Plos One**".** We are grateful to the editor and the reviewers who invested sufficient time to review our manuscript in detail and give constructive and invaluable comments. We have properly incorporated all the comments and suggestions the editor and reviewers provided. All the changes and corrections are included in the manuscript highlighted in yellow, and the revised paper. We have prepared a point-by-point response to all the comments and suggestions given by the reviewers. The revisions have been approved by all the authors. We hope this revised manuscript will better suit the Journal of “Plos One”.

Thank you for your kind consideration,

Sincerely

Zenebe Terefe (on behalf of the co-authors)

College of Agriculture and Environmental Sciences

Bahir Dar University, Bahir Dar, Ethiopia

[zenebegebreyesus@gmail.com](mailto:zenebegebreyesus@gmail.com)

**Responses to Editor Comments**

***01. Comment*:** Please ensure that your manuscript meets PLOS ONE's style requirements, including those for file naming. The PLOS ONE style templates can be found,

***01. Response***: Thank you for your constructive and invaluable comments. We have properly organized the manuscript following the guidelines of PLOS ONE “'s style requirements.

***02. Comment*:** In your Methods section, please provide additional information regarding the permits you obtained for the work. Please ensure you have included the full name of the authority that approved the field site access and, if no permits were required, a brief statement explaining why.

***02. Response***: Thank you for your constructive comments.

Bahir Dar University Research and Publication Directorate authorized the present study to conduct the field experiment and access the field site. Farmers agreed to conduct field trials and collect the soil samples in the study area, as the study has no harmful effects on humans.

***03. Comment***: We note that your Data Availability Statement is currently as follows: [no]

Please confirm at this time whether or not your submission contains all the raw data required to replicate the results of your study. Authors must share the “minimal data set” for their submission.

***03. Response***: Thank you for your constructive comments. All the required data are presented within the manuscript and its Supporting Information files.

***04. Comment***: PLOS requires an ORCID iD for the corresponding author in Editorial Manager on papers submitted after December 6th, 2016. Please ensure that you have an ORCID iD and that it is validated in Editorial Manager.

***04. Response***: Thank you for your comments and I have already an ORCID (0009- 0006-6787-6420).

***05. Comment***: We note that you have referenced

(4. Haile, H., Asefa, S., Regassa, A., Demssie, W., Kassie, K. & Gebrie, S. (2017). Extension manual for acid soil management (unpublished report). ATA (ATA), ed.), Addis Ababa, Ethiopia.

19. Bahir Dar Meteorological Stations (BDMS). (2020). Rainfall, minimum and maximum temperature data from 2011-2020 in Banja district Awi zone Ethiopian from Bahir Dar. Unpublished document.)

which has currently not yet been accepted for publication. Please remove this from your References and amend this to state in the body of your manuscript: (i.e. “Bewick et al. [Unpublished]”) as detailed online in our guide for authors

<http://journals.plos.org/plosone/s/submission-guidelines#loc-reference-style>

***05. Response***: Thank you for your invaluable and pertinent comments and we have removed those unpublished references accordingly.

***06. Comment:*** Your ethics statement should only appear in the Methods section of your manuscript. If your ethics statement is written in any section besides the Methods, please move it to the Methods section and delete it from any other section. Please ensure that your ethics statement is included in your manuscript, as the ethics statement entered into the online submission form will not be published alongside your manuscript.

***06. Response***: We kindly accepted the comment and now the ethics statement has appeared in the Method section of our revised manuscript.

***07. Comment***: We note that; Figure 1 in your submission contains [map/satellite] images which may be copyrighted. All PLOS content is published under the Creative Commons Attribution License (CC BY 4.0), which means that the manuscript, images, and Supporting Information files will be freely available online, and any third party is permitted to access, download, copy, distribute, and use these materials in any way, even commercially, with proper attribution. For these reasons, we cannot publish previously copyrighted maps or satellite images created using proprietary data, such as Google software (Google Maps, Street View, and Earth).

We require you to either (1) present written permission from the copyright holder to publish these figures specifically under the CC BY 4.0 license, or (2) remove the figures from your submission:

***07. Response:*** Thank you very much for your constructive comment. Hence, we have removed Figure 1 from the manuscript accordingly as it does not influence the quality of the manuscript.

***08. Comment.*** Please include your tables in your main manuscript and remove the individual files. Please note that supplementary tables (should remain/ be uploaded) as separate "supporting information" files

***08. Response***: Thank you for your invaluable comments and suggestions. We have included the tables within our main manuscript and supplementary tables are uploaded as separate supporting information files.

**Responses to Reviewers' Comments**

**Reviewer #1:**

***09. Comment***: The previous literature is well-documented by the authors. However, the following research articles must be cited in the introduction and methodology section,1. DOI:10.1007/s11104-023-06016-4 2.DOI:10.3390/microorganisms10102078 3. DOI: 10.4236/ojss.2018.82007

***09. Response:*** Thanks for your constructive comments and suggestions. We have read the articles thoroughly and cited them in the introduction section of the revised manuscript.

**Reviewer #2:**

***10. Comment:*** Title: Please replace the word “of’ with the word ‘in’ before Mecha

***10. Response:*** We kindly accepted the comment and now we have replaced replace the word “of’ with the word ‘in’ before Mecha**.** in the revised manuscript.

**Abstract:**

***11. Comment:*** Line 16: replace the word “of’ with the word ‘in’ before Mecha
***11. Response:*** Thank you for your invaluable comments and we have made corrections in the revised manuscript.

***12. Comment:*** Line 18: make the word arrange in the past form

***12. Response*:** Thank you very much for your constructive comment. We have changed the word arrange in the past form (arranged) accordingly.

***13. Comment:*** Please write clearly, about tons and ton; Use the elaborate form of VC and L

***13. Response:*** We kindly accepted the comments and we have made corrections accordingly in the revised manuscript.

***14. Comment:*** Keywords: Use more appropriate words

***14. Response:*** Thank you for your pertinent and useful comment. We have used more appropriate keywords that can highlight the main research findings in the revised manuscript.

**Introduction:**
***15. Comment*:** In the first sentence, use a conjunction to complete the sentence

***15. Response*:** Thanks for your invaluable and constructive comments. We have used a conjunction in the first sentence accordingly.

***16. Comment*:** In the second sentence add an article before the word Ethiopian and leaching

***16. Response*:** Thank you for your crucial and constructive feedback. Hence, we have incorporated an article accordingly in the revised manuscript.

***17. Comment*:** In the third sentence, ‘acidic (pH 4.1-5.5) soils’ write in nature instead of soils.
***17. Response*:** We kindly accepted the comment and thank you for giving constructive comments. We have replaced and included it accordingly in the revised manuscript.

***18. Comment*:** The first and last sentences of the first paragraph (lines 33-38) are almost the same. Make it in one sentence.

***18. Response*:** We have accepted the reviewer's comment. We have merged the first and last sentences and made significant improvements in the revised manuscript.

***19. Comment*:** Line 39: add the scientific name of the crop.

***19. Response*:** Thank you for giving invaluable comments and we have incorporated the scientific name of the crop (*Hordeum distichum* L) in the revised manuscript.

***20. Comment*:** “Although barley is considered to be the fifth-most common, a widely farmed crop in Ethiopia's highlands, it is extremely susceptible to soil acidity” The sentence is not clear, make it clear and simple.

***20. Response*:** We accepted the comments and suggestions. We have rewritten the sentence properly and now it is clear and simple to understand.

***21. Comment*:** Please remove the decimal and give a round figure (Line 42).

***21. Response*:** Thank you for giving invaluable comment. We have removed the decimal and put a round figure only in the revised manuscript.

***22. Comment*:** Always maintain the same unit

***22. Response*:** We accepted the comment and we have used the same unit throughout the document in the revised manuscript.

***23. Comment*:** Line 47: Please use recent information

***23. Response*:** Thank you for your dedication and insightful comments. We have looked for recent information and incorporated it in the revised manuscript**.**

***24. Comment*:** Line 48-49: Rewrite the sentence considering the article, tense, and punctuation

***24. Response*:** Thanks for your insightful observations and feedback. We have gone through the sentences corrected and rewritten in the revised manuscript.

***25. Comment*:** Line 53 please use recent data

***25. Response*:** Thank you for your comments. we have looked for recent data and references incorporated in the revised manuscript.

***26. Comment*:** Line 54-56. Check these sentences “Besides, vermicompost is enriched with soil microbiota such as nitrogen-fixing and phosphorus-solubilizing organisms and provides several essential macro and micronutrients become more available in VC [15]. It includes the ability to increase soil microbial activity by enhancing oxygen availability, regulating soil temperature, improving porosity, infiltration, and nutrient content, growth promoter and boosting crop yield and quality” and writing following grammatical rules

***26. Response*:** Thank you for your constructive and invaluable comments. We have rearranged and substantially improved the sentences in the revised manuscript.

***27. Comment*:** Line 61: Add an article before the word economically

***27. Response*:** Thank you for your constructive comments. We have cited an indefinite article in the revised manuscript**.**
***28. Comment*:** Line 64: Mention maximizing instead of maximize

***28. Response*:** Thank you for giving invaluable comments and we have mentioned maximizing instead of maximize in the revised manuscript.
***29. Comment*:** Line 66: Write have instead of has

***29. Response*:** Thank you for your critical observations and giving useful feedback. We have accepted the comment and write have instead of has in the revised manuscript.

**Materials and Methods:
*30. Comment*:** Please check the sentence “The study site is located between 11°” 29' 30" N and 11° 33' 0" N latitudes and 37° 07' 0" E and 37° 10' 30" E” longitudes”

***30. Response*:** The comment is well accepted and we have rearranged the sentence as follow: The study area is located between 11° 29' 30" N to 11° 33' 0" N Latitudes and 37° 07' 0" E to 37° 10' 30" E Longitudes.

***31. Comment*:** The study was conducted in 2021 and 2022, but the meteorological data was from 2011 to 2020. Please give the data only during the cropping season.

***31. Response*:** Thank you for your invaluable and constructive comments. We have included the meteorological data for the 2021 and 2022 cropping seasons.

***32. Comment*:** Convert the word productions to production

***32. Response*:** We accepted the comment and we have made correction accordingly in the revised manuscript.

***33. Comment*:** Please rewrite the sentence “The major crops type growing the vicinity are wheat, barley, maize, teff and various types of horticulture”

***33. Response*:** Thank you for your invaluable feedback. We have properly rewritten and improved the sentence in the revised manuscript.

***34. Comment*:** Lines 101 and 103: Check the unit of bulk density

***34. Response*:** Thank you for your constructive comments and suggestions. We have used the same pattern for the unit of bulk density in the revised manuscript.

***35. Comment*:** Line 114: Use sowing instead of planting as seed was used for crop production

***35. Response*:** We accepted the comment and now we have used sowing instead of planting.
***36. Comment*:** Please mention the source of N and P fertilizer

***36. Response*:** Thank you for the invaluable and constructive comment. We have incorporated the source of N and P fertilizer in the revised manuscript.

***37. Comment*:** Please explain whether boron fertilizer was used or not because it is related to grain formation

***37. Response*:** Thank you for your remainder. We have not applied boron fertilizer in this study. only vermicompost and lime were applied in combination.

***38. Comment*:** Line 127: convert the word ground to grinded

***38. Response*:** Thank you for your invaluable comments and We have corrected it the revised manuscript.

***39. Comment*:** Line 133: Please read the sentence carefully

***39. Response*:** Thank you for giving constructive comments and suggestions. We have read the sentence carefully and rearranged it accordingly in the revised manuscript.

***40. Comment:*** Follow the same pattern for writing unit

***40. Response*:** We accepted the comments and we have followed the same pattern regarding writing units in the revised manuscript.

***41. Comment*:** Manual separation of the grains from the straws resulted in yield measurements for each plot in tons (kg ha^-1^). Write the sentence clearly

***41. Response*:** We accepted the comments and we have rewritten the sentence clearly in the revised manuscript.

***42. Comment*:** Check the word ‘hectare’ throughout the manuscript

***42. Response*:** Thank you for your constructive comments and suggestions. We have gone through the whole the manuscript and made correction in the revised manuscript.

***43. Comment*:** Line 148: Unify the unit

***43. Response*:** Thank you for giving useful comments and we have properly corrected this accordingly.

**Tables:
*44. Comment*:** For better understanding, use the short name of the treatment instead of the percent and rate of vermicompost and lime. Then elaborate on the short name of the treatment in the table footnote?

***44. Response*:** We have accepted the comments and now we have used the short name of the treatment instead of the percent and rate of vermicompost and lime rate.

***45. Comment*:** Synchronize the treatment serials in table-1 with table-3,4 and 6**??**

***45. Response*:** Thank you for your invaluable comments and suggestions. We have aligned the treatment serials in Table 1 with Tables 3,4 and 6**.**

***46. Comment:*** Mention the area for vermicompost and lime rate**?**

***46. Response*:** Thank you very much for giving constructive comments. Vermicompost and lime rates are estimated for one hectare. Then, Vermicompost and lime rates required for each experimental plot (6m^2^) was calculated and applied.

***47. Comment*:** Please solve the problem of t/ha, t ha-1, ton/ha, t ha-1, tons, and ton

***47. Response*:** Thank you for your critical observations and giving invaluable comments. We have gone through the manuscript and properly solved inconsistent writing of t/ha, t ha-1, ton/ha, t ha-1, tons, and ton in the revised manuscript.

***48. Comment*:** Please check the sentence “Table 2. Characteristics the study soil and vermicompost before planting” and correct the sentence

***48. Response*:** Thank you for your valuable comments and insightful observations. We have rearranged Table 2 heading (Characteristics of soil and vermicompost before sowing”) in the revised manuscript.

***49. Comment*:** Table-1: Explain, for treatment combination, why don’t use 50% sole lime whether use 50% sole vermicompost? use 150% sole lime but why not 150% vermicompost? also use a combination of 150% VC+100 L but why not 150% L+100% VC

***49. Response*:** Thank you very much for your insightful observation and comments. The treatments were set based on the economic point of farmers, and availability of inputs in the area. Hence, we didn’t use 50% sole lime in this trial since this small lime rate alone cannot be sufficient for optimal crop production based on the value of exchangeable acidity laboratory result. Besides, we didn’t use 150% vermicompost (VC) alone as smallholder farmers cannot prepare such a huge amount of VC due to limited supply of organic inputs and bulkiness. Besides, vermicompost was applied based on recommended nitrogen equivalency (69 N kg ha^-1^) for malt barley production in the study area. To fulfill the recommended amount of nitrogen, the required amount of vermicompost was 5.51 tons ha^-1^. Beyond this it would not be cost-effective for smallholder farmers. **VC?** But we already used 150% L + 100% VC, not 150 % VC + 100% L.

***50. Comment*:** Table-2: BD appears very low, whether the soil is clay and organic carbon content is very low

***50. Response*:** Thank you for giving invaluable comments. As you rightly stated, BD of the soil showed very low although soil organic carbon content is very low**.** The reasons for very low BD could be continuously plowing of experimental plot for crop production results in finer soil particles, optimal porosity. Besides, the cropland is not affected by compaction.

***51. Comment*:** Table 3: Check the p-value of available Phosphorus

***51. Response*:** Thank you for your critical observations and giving constructive comments. We have checked it carefully and corrected properly in the revised manuscript.

***52. Comment*:** Table-6: Please use a round figure for NB and MRR, mention GNR in the table caption whether used GNB in the table footnote and write the correct one

***52. Response*:** Thank you for your insightful observation and invaluable comments. We have used round figures for NB and MRR values in the revised manuscript. Besides, we have mentioned the correct term, GNR not GNB, which is due to typing error

***53. Comment*:** Unify the tables heading with the description heading in the result and discussion section

***53. Response*:** Thank you for giving constructive comments. Now we have aligned the tables heading with the description heading in the result and discussion accordingly.

***54. Comment*:** Please use the same font for the footnote

***54. Response*:** We have accepted the comments. We have used the same font for the footnote of all tables in the revised manuscript.

**Result and discussion**

***55. Comment*:** Please add the field capacity data as irrigation was supplied based on the field capacity

***55. Response*:** Thank you for giving constructive comments. We have added the field capacity data in the revised manuscript accordingly since we have determined the soil water content at field capacity and permanent wilting point before sowing.

***56. Comment*:** 183-184 line: C: N and C/N, write this in a uniform pattern

***56. Response*:** Thank you a lot for your insightful comments and suggestions. We have written the C: N and C/N and other abbreviations in a uniform pattern in the revised manuscript.

***57. Comment*:** Please remove clumsiness and duplication during the writing of results and discussion of results. Discuss the results specifically. Omit unnecessary results.

***57. Response*:** Thank you very much for giving constructive comments. We have rewritten and revised the results and discussion part properly and removed unnecessary duplication in the revised manuscript.

***58. Comment*:** Please reduce the number of references and use recent references, make space for two or more references in the third bracket

***58. Response*:** Thank you very much for giving invaluable and constructive comments. We have reduced the number of references and used recent references in the revised manuscript.

***59. Comment*:** Make similarities for writing p, ap, phosphorus, and available phosphorus

***59. Response*:** We have accepted your insightful comments. We have used similar pattern for writing abbreviations in the revised manuscript.

***60. Comment*:** Line 264: Place a verb after the reference number 43

***60. Response*:** Thank you very much for giving invaluable comments and we have made corrections in the revised manuscript.

***61. Comment*:** Line 276: Put a comma before respectively

***61. Response*:** We have accepted the comments. We have put a comma before respectively in the revised manuscript.

***62. Comment*:** Line 303: Remove the bracket before 29.63

***62. Response*:** Thank you for your critical observation and comment. We have removed the bracket before 29.63 in the revised manuscript.

***63. Comment*:** Please add the value of the control treatment during the comparison
Partial budget analysis:

***63. Response*:** Thank you for your constructive the comment. We have included the value of the control in the revised manuscript.

***64. Comment*:** First sentence (lines 326-329), please read the sentence carefully and rewrite the sentence for a better understanding

***64. Response*:** Thank you for your invaluable comment. We have read the sentence carefully and rewrite the sentence and now it is clear and simple for understanding.

***65. Comment*:** Line 330: Please use a round figure for data value

***65. Response*:** Thank you for your critical observation and useful feedback. We have made corrections in the revised manuscript accordingly.

***66. Comment*:** Please check the net benefit value described in the abstract, partial budget analysis, and conclusion chapters and make uniform

***66. Response*:** Thank you for your insightful observations and constructive suggestions. We have checked the net benefit value in the whole manuscript section and made it uniform in the revised manuscript.

**Conclusion:**

***67. Comment*:** Consider the same pattern for RL, LR, and L

***67. Response*:** Thank you very much for your pertinent comments. We have used the same pattern for RL, LR, and L **throughout** the manuscript.

***68. Comment*:** Line 344: lime compost? not clear

***68. Response*:** Thank you for your insightful observation and comment. It is due to a typing error and now we have corrected it in the revised manuscript.

**References:
*69. Comment*:** References were written whimsically. Rewrite the reference chapter and follow a pattern for all references.

***69. Response*:** The comment is pertinent and constructive. Thus, we have rewritten the all references properly following a uniform format as per the journal guideline.

**Reviewer #3:**

***70. Comment*:** There are minor omissions of some prepositions.

***70. Response*:** Thank you for your insight full observation and comments. We have thoroughly incorporated omitted prepositions.

***71. Comment*:** "The file figures could not be opened because there are problems with the contents" was the message I got when I attempted to view the figures. Please re-upload the file figures.

***71. Response*:** Thank you for your suggestion and feedback. We have reconstructed and reuploaded the file figure.
