# Supplementary material for: Effects of vermicompost and lime on acidic soil properties and malt barley (Hordeum Distichum L.) productivity in Mecha district, northwest Ethiopia
Source: PLoS One. 2024 Dec 6;19(12):e0311914. doi: 10.1371/journal.pone.0311914 (PMC11623467; doi:10.1371/journal.pone.0311914)
Supplement: Attachment — Submitted filename: PLOSONE.....docx [file pone.0311914.s006.docx]

**Comments**

I have gone through the revision of the article on “**Effects of vermicompost and lime on soil properties and malt barley (Hordeum distichum L.) productivity on acidic soils of Mecha district, northwest Ethiopia**”. The following research articles are needed to be cited in the introduction and methodology section before publication.

1. DOI: 10.3390/microorganisms10102078
2. DOI: 10.4236/ojss.2018.82007
