# Supplementary material for: Effects of vermicompost and lime on acidic soil properties and malt barley (Hordeum Distichum L.) productivity in Mecha district, northwest Ethiopia
Source: PLoS One. 2024 Dec 6;19(12):e0311914. doi: 10.1371/journal.pone.0311914 (PMC11623467; doi:10.1371/journal.pone.0311914)
Supplement: Attachment — Submitted filename: Response to the reviwers.docx [file pone.0311914.s007.docx]

Dr. Ravinder Kumar,
Academic Editor,

PLOS ONE

**Subject: Response to Reviewers Comments**

Dear Dr. Ravinder Kumar,

We want to thank you for allowing us to resubmit the revised manuscript entitled "**Effects of vermicompost and lime on soil properties and malt barley (*Hordeum distichum* L.) productivity on acidic soils in Mecha district, northwest Ethiopia** [PONE-D-24-13572] for publication in Plos One". We are grateful to the editor and the reviewers who invested sufficient time to review our manuscript in detail and give constructive and invaluable comments. We have properly incorporated all the comments and suggestions the editor and reviewers provided. All the changes and corrections are included in the manuscript track change highlighted, and the revised paper. We have prepared a point-by-point response to all the comments and suggestions given by the reviewers. The revisions have been approved by all the authors. We hope this revised manuscript will better suit the Journal of “Plos One”.

Thank you for your kind consideration,

Sincerely

Zenebe Terefe (on behalf of the co-authors)

College of Agriculture and Environmental Sciences

Bahir Dar University, Bahir Dar, Ethiopia

[zenebegebreyesus@gmail.com](mailto:zenebegebreyesus@gmail.com)

**Responses to Editor Comments**

01. ***Comment*:** Please review your reference list to ensure it is complete and correct. If you have cited papers that have been retracted, please include the rationale in the manuscript text, or remove these references and replace them with relevant current references.

***01. Response*:** Thank you for providing constructive and relevant comments. We have carefully reviewed the reference lists and properly revised and corrected them based on the journal reference guidelines. Some old references are removed and replaced with relevant and recent ones. Besides, we have not cited retracted articles in the manuscript.

**Responses to Reviewers' Comments**

**Reviewer #1:**

***02. Comment***: The following research articles need to be cited in the introduction and methodology section before publication:

1. DOI: 10.3390/microorganisms10102078

2. DOI: 10.4236/ojss.2018.82007:

***02. Response:*** Thank you for your constructive suggestions. We have read both articles thoroughly and cited the articles in the discussion section of the revised manuscript.

**Reviewer #2:**

***03. Comment:*** Read the title and reference chapter carefully.

***03. Response:*** Thank you for your invaluable and constructive comments. We have read the title and reference part critically, and we made significant revisions to the title and references in the revised manuscript accordingly.

***04. Comment:*** Add malt barely in place of integrated with keywords

***04. Response:*** Thank you so much for your useful feedback and now we have incorporated malt barley instead of integrated in the revised manuscript.

***05. Comment:*** still a problem with the old reference in the introduction chapter

***05. Response*:** Thank you for your constructive comments. We have removed old references and replaced them with more recent references in the revised manuscript.

***06. Comment:*** still the problem of L, RL, t ha-1, t/ha, tons ha-1

***06. Response:*** Thank you for your insightful observations and relevant comments. We have carefully and consistently rewritten all units and abbreviations in the revised manuscript.

***07. Comment:*** Please define it or explain it properly ETB

***07. Response:*** Thank you very much for your invaluable comment and we kindly accepted the comment. We have clearly defined ETB as Ethiopian Birr first mentioned before the abbreviation in the revised manuscript.

***08. Comment*:** Please have a careful look the lines, 75-77, 127, 133, 190, 247, 251, 254, 269, 275, 303, 333

***08. Response*:** Thank you for your constructive and pertinent comments. We have made a careful review of the lines mentioned above and properly rearranged and improved the sentences.

***09. Comment*:**  Remove the clumsiness and duplication of writing in the discussion of soil properties

***09. Response*:** Thank you very much for your invaluable comments. We have carefully read the manuscript and removed unnecessary literature and duplication of writing in the discussion of soil properties in the revised manuscript

***10. Comment*:**  Please go through many rounds of editing regarding typos and grammatical error

***10. Response*:** Thank you for reading of the manuscript in-depth and giving useful comments. Thus, we have read the manuscript carefully and properly addressed the typos and grammatical errors.

***11. Comment*:** Need improvement of language in result and discussion writing

***11. Response*:** Thank you very much for your pertinent comments and feedback. Now we have made significant language improvements in result and discussion writing.

**Reviewer #3:**

***12. Comment*:** Line 22- please define VC and L at first mention before the abbreviation
***12 Response*:** Thank you for your constructive and insightful comments. We have properly defined VC as vermicompost and L as lime as first mentioned in the revised manuscript.

***13. Comment*:** Line 86- What is NPS? Define first at first mention before abbreviation. Farmers widely apply.... delete used to

***13. Response*:** Thank you very much for your invaluable comments. We have defined NPS as a Nitrogen-Phosphorus-Sulfur blend fertilizer at first mention before the abbreviation. Besides, we deleted the words used in the revised manuscript accordingly.

***14. Comment*:** Line 108- Define FC, PWP

***14. Response*:** Thank you for your critical observations and for giving pertinent feedback. Now we have defined FC(field capacity) and PWP (permanent wilting point) in the revised manuscript.

***15. Comment*:** Line 165- Is there any reference to the economic analysis assumption?***15. Response*:** Thank you for raising invaluable comments. Yes, there is a reference to the economic analysis assumption. The assumption stated that grain and straw yields produced from research sites need to be adjusted down by 10% to narrow the yield gap between yields obtained from experimental plots and farmers’ fields. Hence, economic analysis was done based on the method provided by CIMMYT (1988).

***16. Comment*:** Line 281- Change to In line with the report of (54), an increase...
***16. Response*:** Thank you for your useful comments and we have made corrections in the revised manuscript accordingly.

***17. Comment*:** Line 301-What is RL? Let there be consistency in abbreviation. RL are indicated in some parts, L in others, and Lime elsewhere. Do these represent the same thing? Please review.

***17. Response*:** Thank you for your invaluable and constructive comments. We kindly accepted the comment, and now we have used L (lime) instead of RL(recommended lime) throughout the revised manuscript.

***18. Comment*:** Line 332- The application of...

***18. Response*:** Thank you for giving pertinent comments.. We have properly rewritten the sentence in the revised manuscript.

***19. Comment*:** Line 370- 'Provided time' how? If this means permitting the researchers or giving time off to the researchers to conduct the research, please indicate as such so the sentence will not be ambiguous.

19***. Response*:** Thank you very much for your insightfull observation and feedback. The meaning of this sentence is that I thank the Amhara Regional Bureau of Agriculture and Woreta College of Agriculture for permitting me to pursue my Ph.D. studies. Now we have rearranged and improved this sentence in the revised manuscript.
